# Temporal and Spatial Denoising of Depth Maps

**DOI:** 10.3390/s150818506

**Published:** 2015-07-29

**Authors:** Bor-Shing Lin, Mei-Ju Su, Po-Hsun Cheng, Po-Jui Tseng, Sao-Jie Chen

**Affiliations:** 1 Department of Computer Science and Information Engineering, National Taipei University, New Taipei City 23741, Taiwan; 2 Department of Biomedical Engineering, Yuanpei University of Medical Technology, Hsinchu 30015, Taiwan; E-Mail: merri1024@gmail.com; 3 Department of Software Engineering and Management, National Kaohsiung Normal University, Kaohsiung 82444, Taiwan; E-Mail: cph@nknu.edu.tw; 4 Graduate Institute of Electronics Engineering, National Taiwan University, Taipei 10617, Taiwan; E-Mails: r01943155@ntu.edu.tw (P.-J.T.); csj@ntu.edu.tw (S.-J.C.)

**Keywords:** depth image, spatial-temporal denoising, RGB-D sensor, hole padding

## Abstract

This work presents a procedure for refining depth maps acquired using RGB-D (depth) cameras. With numerous new structured-light RGB-D cameras, acquiring high-resolution depth maps has become easy. However, there are problems such as undesired occlusion, inaccurate depth values, and temporal variation of pixel values when using these cameras. In this paper, a proposed method based on an exemplar-based inpainting method is proposed to remove artefacts in depth maps obtained using RGB-D cameras. Exemplar-based inpainting has been used to repair an object-removed image. The concept underlying this inpainting method is similar to that underlying the procedure for padding the occlusions in the depth data obtained using RGB-D cameras. Therefore, our proposed method enhances and modifies the inpainting method for application in and the refinement of RGB-D depth data image quality. For evaluating the experimental results of the proposed method, our proposed method was tested on the Tsukuba Stereo Dataset, which contains a 3D video with the ground truths of depth maps, occlusion maps, RGB images, the peak signal-to-noise ratio, and the computational time as the evaluation metrics. Moreover, a set of self-recorded RGB-D depth maps and their refined versions are presented to show the effectiveness of the proposed method.

## 1. Introduction

The availability of low-cost RGB-D (depth) sensors has helped expand numerous research fields such as image processing [1], 3D printing [2], surveillance [3], object tracking [4], and the computer vision of robotics [5]. Previously, many other types of cameras were used to generate depth maps, namely time of flight (TOF), light detection and ranging (LIDAR), and stereo cameras [6], and each had a different principle of operation. However, they were substantially more expensive and occupied a large volume. By contrast, low-cost RGB-D sensors are handheld devices that are easy to set up and affordable. Examples of these low-cost depth sensors are Microsoft’s Kinect and Asus’s Xtion PRO LIVE. These sensors have been widely used in the aforementioned applications because of their high performance [6].

However, the problems associated with these sensors are the presence of noise or outliers and the instability of depth maps in the spatial domain, which reduce the performance of the depth camera in applications. Annoying outliers appearing in depth images contaminate the depth information, and the depth pixel value obtained in the spatial domain is inaccurate.

Solving the noise problem is crucial because noise affects applications such as 3D reconstruction and depth data compression by using an RGB-D camera [7]. Although there have been many studies on the applications of these depth sensors, few have focused on the problems inherent to these sensors [8].

RGB-D sensors such as Microsoft Kinect and Asus Xtion PRO LIVE have many problems with regard to the generated depth images: noise, inaccurate depth pixel values, and temporal vibrations. Errors in the sensors are usually caused by the inadequate calibration of sensors and the inaccurate measurement of disparities [1] between sensors. They are also related to the measurement setup, such as light conditions and image geometry (distance between the objects and the sensors, and the orientation of the objects). In a very bright environment, depth images recorded using RGB-D sensors cannot form the speckle pattern of the projected object because of the low contrast between the infrared (IR) pattern and bright light [9]. In addition to the sensors and environment, the properties of the objects also affect the depth images. A shiny or reflective surface of an object appears overexposed in an infrared image and leads to error disparities.

Many studies have been conducted on the errors appearing in RGB-D depth images. Some studies have used traditional approaches involving denoising algorithms, for example, the bilateral filtering algorithm [10]. A modification of bilateral filtering was proposed in [7], which also proposed divisive normalized bilateral filtering (DNBL) for achieving temporal and spatial filtering. Some studies were conducted to improve the quality of depth images; a stereo matching method that prevents holes was proposed in [11,12]. Studies on temporal filtering were presented in [8,13]. The two approaches presented in [8,13] involve temporal filters with adaptive thresholds that filter out unreasonable depth values and smooth the temporal vibration. In [14], a fusion-based inpainting method was proposed to improve the quality of the depth map. However, these approaches ignore spatial filtering, which leads to undesirable values in depth images. A few approaches combining temporal and spatial filtering have been proposed. In [7], Fu *et al.* proposed a method to fill the holes in depth images in the spatial domain, and applied a modified bilateral filter to reduce vibrations in the temporal domain. However, the prehole filling of the authors’ assumption and blurry image leads to inaccuracy of depth pixel values. Furthermore, the performance has not been proven because of the lack of quantitative data. In [15], the authors proposed a temporal and spatial denoising filter algorithm to enhance the quality of depth images by using RGB images. The algorithm first determines the median of the pixel values in the temporal domain. It then fills the holes in the depth image by using a median filter and several thresholds. However, this method relies on many thresholds and therefore is not adaptive. Moreover, the use of RGB images for denoising has a drawback; an example is a scene with different depth measurements but with objects of the same color or objects in a dark environment. The method does not provide quantitative information about the depth accuracy of pixel values. It is difficult to distinguish the quality of depth images solely from visual information. In [16], a depth color fusion method was proposed. The method could efficiently reduce distance-dependent depth maps, spatial noise, and temporal random fluctuations. However, the method requires processing both depth and color images, including foreground and background segmentation; thus, it may cost more hardware requests such as parallel implementation with a graphics processing unit architecture or cloud computing.

To remove the depth image noise, obtain accurate depth values, and eliminate depth instability in the temporal domain, a method based on the modification of exemplar-based inpainting, which was originally proposed in [17], is introduced in this paper. In [17], Criminisi *et al.* proposed a method to fill holes that were removed previously. The method first assigns priorities to pixel locations ready to be filled. Second, according to the priority, the pixel or area is filled by referring to neighboring pixels or a macroblock. The method is used to inpaint the region or object removed an RGB image. In [18], another method based on the modification of exemplar-based inpainting was proposed. However, this method does not provide the optimal combination of parameters and an evaluation of performance, and ignores spatial filtering.

Our study, based on the method in [17], is similar to the concept of filling holes or outliers in depth images. Thus, the proposed method can be used to fill holes, increase the accuracy of depth values, and smooth temporal vibrations for enhancing the quality of depth images generated using RGB-D sensors.

## 2. Methodology

As mentioned in the previous section, depth maps generated using depth sensors show noise or outliers, inaccurately measured depth distances, and unstable depth values in the temporal domain. To overcome these problems, a method based on exemplar-based inpainting, which was presented in [17], is proposed. The proposed method includes a modified exemplar-based inpainting method and a temporal filter to pad outliers, correct inaccurate pixels, and stabilize the temporal variation in the pixel values in depth maps.

**Figure 1:**
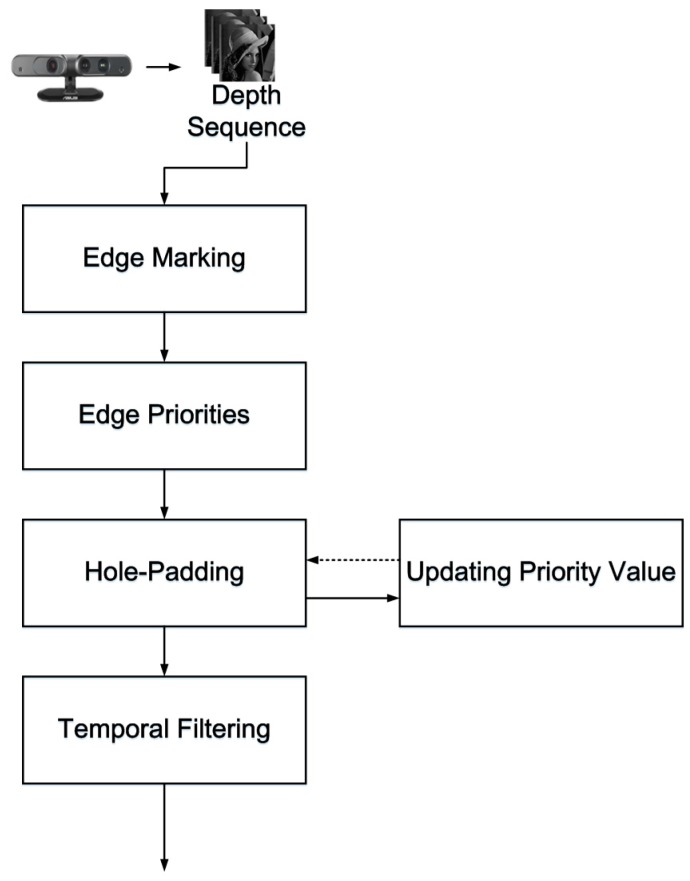
System architecture of the proposed method.

The system architecture is shown in Figure 1. There are five steps in the processing of a noise-containing depth sequence: (1) edge marking; (2) assigning edge priorities; (3) hole padding; (4) updating priority values; and (5) temporal filtering. Steps 1 to 4 are based on a modified version of the exemplar-based inpainting method, with the modification being performed for application to depth map filtering.

### 2.1. System Architecture

**Figure 2:**
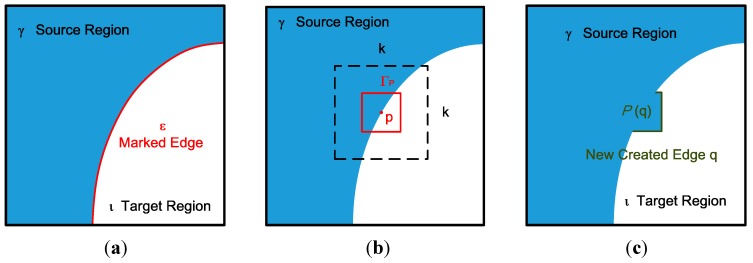
(**a**) Edge marking; (**b**) search range in the hole-padding process; (**c**) patch pasting in the hole-padding process.

In Figure 1, an RGB-D sensor generates depth sequences and color sequences, but only the depth sequences are input. In the edge marking step, the system marks the edges around the holes or outliers, and these marked edges are the target pixels chosen to be padded first, as shown in Figure 2a. In Figure 2a, ε denotes the marked edge (red line), ι denotes the target region (hole region), and γ denotes the source region (holeless regions).

In the edge priority step, the pixels of the depth map, which are the edges marked in the edge marking step, are assigned priorities according to a certain rule introduced in Section 2.2. As shown in Figure 2a, the priorities are assigned to pixels on the marked edge ε.

In the hole-padding step, holes are padded on the basis of the priorities assigned to the pixels. The padding method involves a comparison of the target patch with a neighboring patch within the search range. The comparison begins with the highest priority pixel located at the center and the search range within [−*n*, +*n*] increased in the x and y directions. The similarity between the target patch and the reference patch is then evaluated and the target areas are replaced with the most similar reference patch, as shown in Figure 2b.

In Figure 2b, the red box denotes the target patch, **p** denotes the center of the patch, and *k* denotes the search range. Figure 2c shows the white and blue areas in the red macroblock of Figure 2b padded with a reference patch from the blue areas.

After the target areas are replaced with the reference patch, new edges and holes emerge. As shown in Figure 2c, the green edges are the newly created edges that have no priorities. Therefore, in the updating priority values step, the green edges are assigned priorities, and the hole-padding step is then executed repeatedly until all the hole areas (target regions) are padded with valid values. In the last temporal filtering step, pixels are filtered to eliminate temporal variation in the pixel base. Details of each step are provided in the following sections.

### 2.2. Edge Priorities

The edge marking process is the same as that in [17]. After edge marking, the edge priorities process assigns priorities to all the edges marked in the edge marking process. The priority assignment was modified on the basis of exemplar-based inpainting by using an additional weighting scheme to improve the depth image quality. The priority is computed and assigned for every patch on the boundary, and the priority function is shown in Equation (1).

Given a point **p** that is the center of patch Γ_p_ for some **p∈**ε (Figure 2b), the priority *P*(**p**) is defined as the product of three terms:

*P*(**p**) = *C*(**p**)*D*(**p**)*R*(**p**) (1)

where *C*(**p**) denotes the *confidence* term, *D*(**p**) represents the *data* term, and *R*(**p**) denotes the *right-first* term. *C*(**p**) and *D*(**p**) are the same as those in [17]. *R*(**p**) is the weighting term, and it is based on the depth image IR shadow phenomenon. In this study, the setting of *R*(**p**) in Equation (2) is based on experiments. Noise typically appears in two parts in a scene: (1) in the body of the object and (2) around the right part of the object’s silhouette. One of these parts should be removed first. In *R*(**p**), (2) is processed first. The following functions describe how *R*(**p**) works:

(2) $$R(p)=\;\left\{ \begin{array}{l} \;\frac{2}{3},\;if\;LH(p)\geq RH(p)\\ \;\frac{1}{3},otherwise \end{array}\right.$$

(3) $$LH(x,y)=\;\sum_{i=0}^{n}\sum_{j=0}^{\frac{n}{2}}\;F(x+j,\;y+i)\;$$

(4) $$RH(x,y)=\;\sum_{i=0}^{n}\sum_{j=\frac{n}{2}}^{n}\;F(x+j,\;y+i)\;$$

(5) $$F(x,y)=\;\left\{ \begin{array}{l} \;1,\;if\;I(x,y)\;=\text{0}\\ \;0,otherwise \end{array}\right.$$

Equations (2)–(5) are explained from the last to the first as follows: I(x, y) is the intensity of position (x, y), and in Equation (5), *I*(*x,y*) = 0 denotes that position (x, y) is a hole. In Equations (3) and (4), n is half of the length or width of patch Γ_p_ (usually the length is equal to the width); F(x, y) indicates whether position (x, y) is a hole (0 pixel intensity denotes holes). *LH*(**p**) refers to the number of holes on the left side.

Assigning higher weights to the area around the right part of the object’s silhouette influences the padding direction in *R*(**p**). In this process, in addition to the previous two weighting terms, *C*(**p**) and *D*(**p**), *R*(**p**) should control the padding flow to pad the area around the object’s silhouette. Because the noise around the object’s silhouette is usually considered as background noise, the background area should be padded instead of the objects. When the noise around the right part of the object’s silhouette is padded, for eliminating the noise in the body of the object, the background area is not considered as the reference; instead, the body area pixels are used as the reference.

### 2.3. Hole Padding

In this step, the holes on the depth map are filled patch by patch. In the edge priorities step, the edge pixels were assigned priorities. On the basis of these priorities, the edge pixel with a higher priority is chosen to start the hole-padding process. To start the process, pixel **p** is set to be the center of patch Γ_p_ and a search is made for the most similar areas in the source region γ, as shown in Figure 2b.

To search for the most similar areas for padding, the patch computes the sum of squared differences with its neighboring patches. This direct sampling source region approach avoids blurry fill-in, which causes blurring in depth maps [15]. The search process is valid in the source region, and it would not be valid in any similar patch that includes hole pixels. The comparing function is defined as:

(6) $${\text{Γ}}_{\text{g}}=\arg\underset{{\text{Γ}}_{\text{q}}\in \gamma }{\min}d({\text{Γ}}_{\text{p}},{\text{Γ}}_{\text{q}})$$

where Γ_q_ denotes the patch centered at **q**. The function compares the target patch Γ_p_ with its neighbor Γ_q_, and, depending on the comparison result, either chooses Γ_p_ as the candidate patch or discards it. The comparison process starts with a certain range within the search range. The search range begins from the center **p** and extends to a distance of k/2 pixel toward the top, left, right, and bottom of **p**. Different search methods provide different advantages in terms of speed or other evaluation metrics. The proposed method adopts a modified three-step search (TSS).

The TSS consists of three steps:

Step 1 An initial step size is chosen. Eight patches at a distance of the step size from the center are chosen for comparisons.
Step 2 The step size is half of the search range in Step 1. The center is moved to the point with the minimum distortion.
Step 3 Steps 1 and 2 are repeated until the step size is smaller than 1.

**Figure 3:**
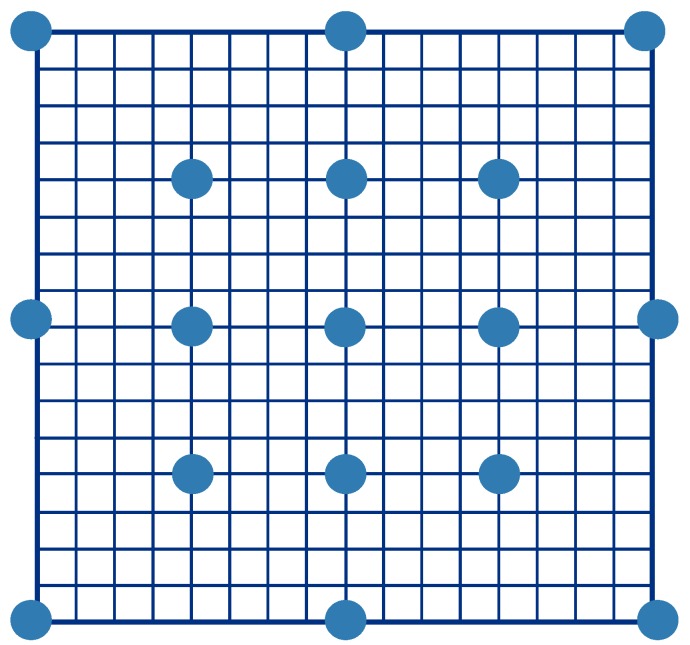
Modified three-step search.

The process of the modified TSS is similar to that of the TSS, but it exhibits enhanced performance. A larger beginning search range for the modified TSS generates more comparisons. In Figure 3, all the blue points indicate the search points, and the search process starts from these points. The eight blue points around the center are extra search points, and such addition does not occur in the traditional TSS. A larger search range is associated with a larger number of search points. For example, in Figure 3, given a 32 × 32 search range, the beginning search range in the TSS is 16 × 16. The beginning search range in the modified TSS is the same as that in the TSS, and the first step of the modified TSS performs 8 × (k/16) + 1 comparisons. For a search range of 32 × 32, the modified TSS performs 8 × (32/16) + 1 = 17 comparisons, as shown in Figure 3. Steps 2 and 3 of the modified TSS are exactly the same as those of the TSS, with the beginning search range being half and a quarter of those of the TSS for Steps 2 and 3, respectively.

The modified TSS was devised by adapting the TSS to our method; for example, in the modified method, when comparing patches with the target patch, any patch containing holes is ignored. If all the patches contain holes, then in Step 1, none of them can be a candidate for comparison with the target patch. Instead, the search range would be increased as the patch comparison restarts. If all the patches are ignored in Step 2, the new target point would be the same as that chosen in Step 1, and the patches would be compared in Step 3.

For updating the priority value, the priority computation is similar to that in [17]. *R*(**p**) is not recomputed to ensure the padding flows toward the padded areas but not around the background. The updating process proceeds until all the edges are considered and holes padded.

### 2.4. Temporal Filtering

The temporal filtering process is performed to prevent the temporal vibration of depth values. In a video sequence, as shown in Figure 1, the pixel values vary with the passage of time. In this step, by applying a temporal filter, the variation of pixel values at a given position is smoothed. The proposed temporal filter in design is also considered the spatial components so that the filter can reduce spatial noise again. The following functions provide details of the temporal filter:

(7) $$I_{t}\left(x,y\right)=\{\begin{array}{c} \sum_{j=0}^{m}\frac{GS_{t}\left(x,y\right)\cdot G\left(t+j\right)\cdot I_{t-j}\left(x,y\right)}{\omega },\;if\;\left(I_{t}\left(x,y\right)-I_{t-1}\left(x,y\right)\right)\leq Th_{∆}\\ I_{t}\left(x,y\right),\;otherwise \end{array}$$

(8) $$GS_{t}\left(x,y\right)=e^{-\frac{x^{2}+y^{2}}{2{\sigma_{s}}^{2}}}$$

(9) $$G\left(t\right)=\frac{1}{\sqrt{2\pi {\sigma_{t}}^{2}}}e^{-\left(\frac{t^{2}}{2{\sigma_{t}}^{2}}\right)}$$

(10) $$\omega =\overset{m}{\underset{j=0}{\sum }}G\left(t+j\right)$$

where G(t) denotes a one-dimensional Gaussian filter, and $GS_{t}\left(x,y\right)$ denotes a two-dimensional Gaussian filter; in Equation (7), *t* denotes the current frame index, $I_{t}\left(x,y\right)$ denotes the current frame pixel intensity, $\;Th_{∆}$ denotes the threshold for the intensity difference between a current frame and a previous frame, and ω denotes the sum of the Gaussian functions; in Equations (8) and (9), ${\text{σ}}_{t}$ and ${\text{σ}}_{s}$ are both the standard deviations.

The function Equation (7) calculates the difference between the current frame and previous frames. To observe whether a pixel value varies in the temporal domain, the threshold is set to a certain value to determine whether the pixel state is moving or static. If the pixel state is moving, the pixel value varies in a large range. Thus, if the range is larger than the threshold, the pixel value would not be replaced by a new value. However, if the pixel between frames is within a small range, it would be interpreted as being in a static state, and the value would be replaced with a value similar to the values of the previous frames. Thus, a new pixel value would be calculated on the basis of Equation (7).

## 3. Experimental Results

Our proposed method was tested on the following two sets of databases: (1) Tsukuba Stereo Dataset [19] and (2) depth maps of real-world scenes generated using Asus Xtion PRO LIVE, and Milani’s Kinect dataset [20]. Because the first dataset provides the ground truth for evaluating the padding results, its peak signal-to-noise ratio (PSNR), structural similarity (SSIM), and computational time can be calculated for comparison with those of the original unpadded depth maps with occlusion. For the second and third dataset, because the ground truth cannot be acquired for comparison with the original depth maps, only the computational time and padded depth maps can be shown for evaluating the performance of the proposed method. The third dataset is from Milani’s Kinect dataset [20]. Milani used two Kinects to perform the environment, the first Kinect generated the cross-talk noise to the second Kinect, and the second Kinect acquired the noisy depth maps. These noisy depth maps were obtained in different included angles (90°, 54°, 36°, 18°, 0°) of two Kinects in order to vary the level of cross-talk noise. The experimental results were compared with those of [7,21] to demonstrate the superiority of the proposed method.

All the experiments were conducted on an Intel(R) Core(TM) i7-3370 3.4 GHz CPU with 16.0 GB of DDR3 DRAM. The programs were implemented in C++ language. The search range and patch were set to different sizes for performance evaluation.

### 3.1. Experiments on the Tsukuba Stereo Dataset

**Figure 4:**
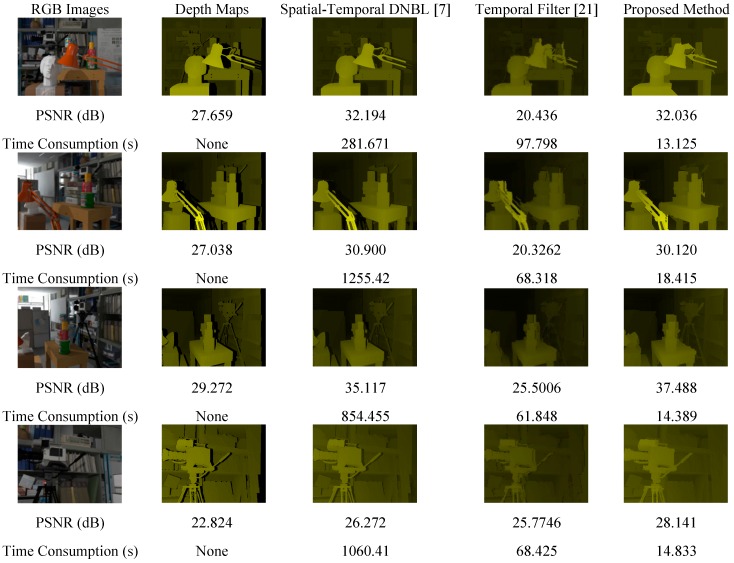

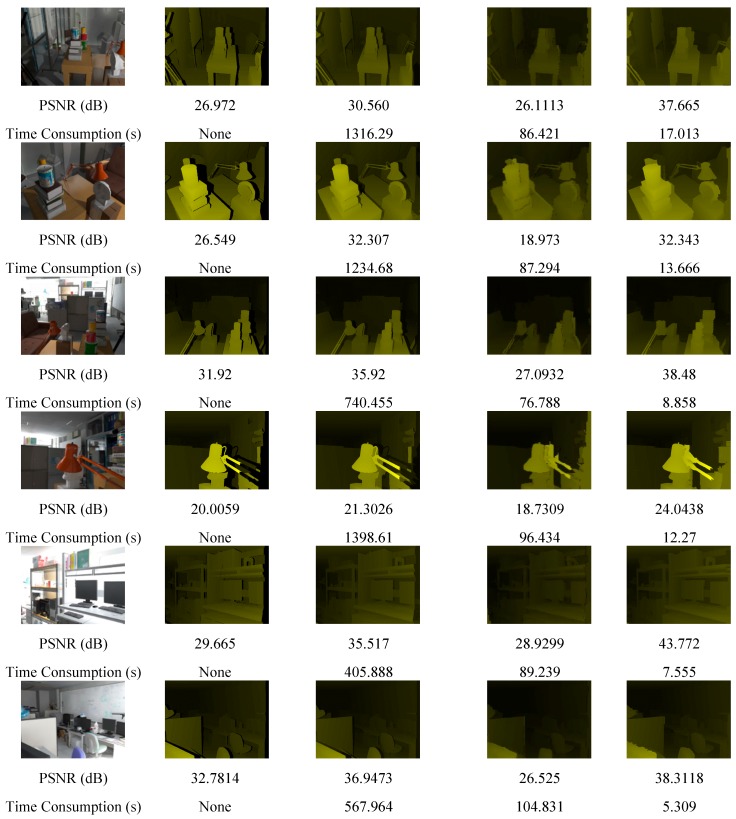
Ten different images from the Tsukuba Stereo Dataset.

The experiments on the Tsukuba Stereo Dataset were divided into two parts. First, ten different images (Figure 4) were chosen from 1800 images, and performance evaluation was performed for different patch sizes and search ranges. Second, comparisons of the experimental results obtained for the ten images were performed for different patch sizes and search ranges in the first step, and the patch size and search range corresponding to the most favorable performance were applied to all 1800 frames. 

The image indices were 1, 509, 481, 214, 291, 347, 459, 525, 715 and 991. These ten images were reconstructed for greater similarity with the ground truth, except for the large hole areas to the right of the scene. All the images were computed in a patch size of 3 × 3, and the search range was 104 × 104. All the PSNR improvements are shown in Figure 4.

**Figure 5:**
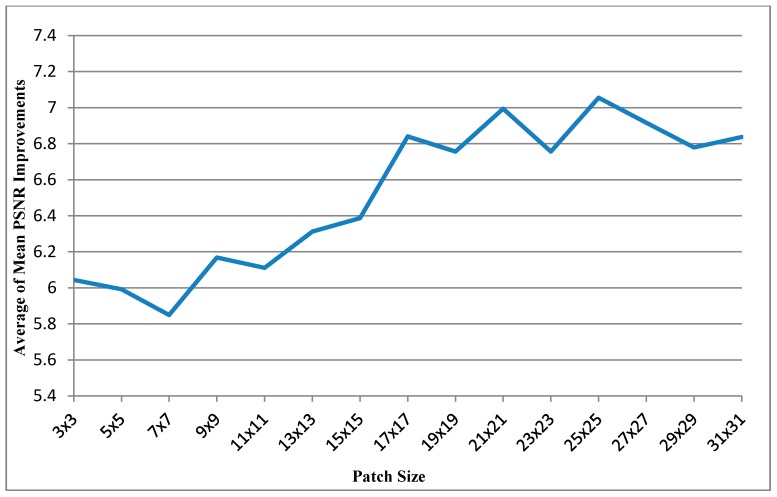
Curve of average of mean PSNR Improvements *vs.* patch size.

**Figure 6:**
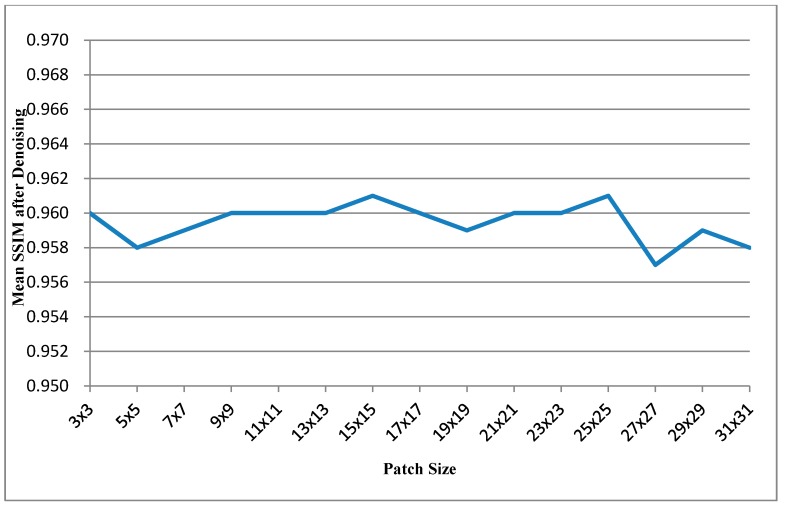
Curve of average of mean SSIMs after denoising *vs.* patch size.

Table 1 shows the results obtained for the ten images for different patch sizes. The leftmost column shows different frame indices. The next four columns show the performance improvements in the PSNR for images with different patch sizes, and each value is the mean of PSNR improvements obtained with different search ranges (from 8 × 8 to 112 × 112). For each frame, the rightmost column shows the patch size corresponding to the most favorable result. Table 2 that has same experimental conditions as Table 1 shows the mean SSIM values after denosing *versus* patch size. And further, the most right columns of Table 1 and Table 2 can be respectively illustrated as Figure 5 and Figure 6. Figure 5 shows the curve of average of mean PSNR Improvements *versus* patch size. Figure 6 shows the curve of average of mean SSIMs after denoising *versus* patch size.

**Table 1:** Mean PSNR improvement *vs.* patch size.

Index											
Patch Size	1	509	481	214	291	347	459	525	715	991	Average
3 × 3	4.006	2.474	7.266	5.252	9.305	2.688	6.252	3.739	13.807	5.652	6.044
5 × 5	4.372	1.332	7.273	5.118	9.860	2.670	5.479	2.568	15.461	5.787	5.992
7 × 7	2.890	1.595	7.673	5.344	8.871	3.209	4.058	3.516	16.059	5.275	5.849
9 × 9	2.884	1.468	7.302	5.185	9.607	5.380	4.590	3.423	16.136	5.705	6.168
11 × 11	2.869	0.925	7.394	5.024	10.058	5.262	5.010	3.161	13.807	7.601	6.111
13 × 13	2.508	0.994	7.520	5.002	10.843	5.573	5.161	3.231	14.517	7.774	6.312
15 × 15	2.822	1.103	7.722	5.084	10.777	4.461	4.787	3.473	13.825	9.812	6.387
17 × 17	2.499	0.228	7.846	5.262	10.501	5.559	4.249	3.590	18.118	10.543	6.840
19 × 19	2.611	0.461	7.669	5.204	11.464	5.224	4.797	3.623	16.403	10.100	6.756
21 × 21	2.780	0.941	8.301	5.213	9.926	5.987	4.885	3.210	18.397	10.295	6.994
23 × 23	3.033	0.984	7.701	4.972	10.818	5.277	4.250	3.133	16.272	11.121	6.756
25 × 25	2.924	0.270	8.525	5.080	10.442	5.417	5.880	3.900	16.707	11.406	7.055
27 × 27	2.468	0.433	7.180	5.032	10.636	5.037	5.971	3.721	17.395	11.285	6.916
29 × 29	2.825	−0.485	8.651	5.104	10.105	5.795	4.690	3.414	17.638	10.049	6.779
31 × 31	3.334	0.536	7.624	5.130	9.864	5.013	5.467	2.996	17.705	10.697	6.837
The Best	5 × 5	3 × 3	29 × 29	7 × 7	19 × 19	21 × 21	3 × 3	25 × 25	21 × 21	25 × 25	25 × 25

**Table 2:** Mean SSIM after denoising *vs.* patch size.

Index											
Patch Size	1	509	481	214	291	347	459	525	715	991	Average
3 × 3	0.950	0.933	0.970	0.969	0.970	0.942	0.977	0.916	0.993	0.981	0.960
5 × 5	0.948	0.932	0.969	0.968	0.970	0.939	0.975	0.908	0.995	0.980	0.958
7 × 7	0.948	0.928	0.970	0.968	0.967	0.944	0.971	0.916	0.995	0.982	0.959
9 × 9	0.944	0.928	0.971	0.968	0.969	0.953	0.972	0.916	0.995	0.984	0.960
11 × 11	0.944	0.926	0.971	0.965	0.970	0.956	0.972	0.911	0.994	0.987	0.960
13 × 13	0.942	0.925	0.971	0.965	0.974	0.956	0.972	0.912	0.994	0.989	0.960
15 × 15	0.944	0.931	0.971	0.964	0.974	0.956	0.972	0.913	0.994	0.992	0.961
17 × 17	0.940	0.925	0.972	0.965	0.972	0.956	0.969	0.913	0.996	0.992	0.960
19 × 19	0.938	0.920	0.971	0.963	0.977	0.958	0.970	0.911	0.994	0.991	0.959
21 × 21	0.941	0.921	0.973	0.963	0.975	0.962	0.971	0.911	0.995	0.992	0.960
23 × 23	0.942	0.922	0.971	0.962	0.974	0.959	0.968	0.911	0.994	0.993	0.960
25 × 25	0.942	0.914	0.975	0.965	0.974	0.963	0.973	0.911	0.994	0.993	0.961
27 × 27	0.936	0.912	0.968	0.962	0.972	0.959	0.972	0.902	0.994	0.993	0.957
29 × 29	0.941	0.911	0.974	0.964	0.972	0.964	0.971	0.907	0.995	0.991	0.959
31 × 31	0.942	0.913	0.969	0.964	0.972	0.959	0.972	0.903	0.994	0.992	0.958
The Best	3 × 3	3 × 3	25 × 25	3 × 3	19 × 19	29 × 29	3 × 3	9 × 9	17 × 17	27 × 27	25 × 25

In Table 3 and Table 4, the mean computational time (in seconds) is listed for different search ranges. All the columns are arranged similarly to those of Table 1. Table 5 shows the results of comparisons between different search ranges. The leftmost column shows the search range (*n* × *n*). The next four columns show the mean PSNR improvement of the ten images for different patch sizes. The rightmost column shows the rank of the search ranges and the most favorable one is presented in boldface. Table 6 that has same experimental conditions as Table 5 shows the mean SSIM values after denosing *versus* search range.

**Table 3:** Mean computational time *vs.* small patch size.

Patch Size								
Search Range	3 × 3	5 × 5	7 × 7	9 × 9	11 × 11	13 × 13	15 × 15	17 × 17
8	14.894	7.220	5.369	4.461	4.215	4.209	4.233	4.367
16	15.034	7.305	5.426	4.545	4.274	4.210	4.208	4.292
24	15.166	7.387	5.522	4.534	4.292	4.256	4.255	4.340
32	15.303	7.460	5.473	4.561	4.374	4.306	4.301	4.387
40	15.425	7.537	5.367	4.568	4.397	4.360	4.352	4.438
48	15.596	7.611	5.417	4.676	4.486	4.418	4.410	4.492
56	15.767	7.697	5.506	4.690	4.510	4.473	4.471	4.553
64	15.937	7.751	5.596	4.800	4.569	4.534	4.526	4.610
72	16.094	7.860	5.590	4.811	4.626	4.592	4.587	4.671
80	16.268	7.957	5.689	4.917	4.682	4.648	4.651	4.733
88	16.431	7.902	5.653	4.939	4.744	4.708	4.711	4.791
96	16.636	7.982	5.797	5.044	4.808	4.770	4.764	4.857
104	16.812	8.087	5.834	5.061	4.861	4.828	4.827	4.914
112	16.970	8.039	5.853	5.122	4.922	4.887	4.890	4.978

**Table 4:** Mean computational time *vs.* bigger patch size.

Patch Size							
Search Range	19 × 19	21 × 21	23 × 23	25 × 25	27 × 27	29 × 29	31 × 31
8	4.481	4.585	4.735	4.964	5.128	5.352	5.468
16	4.399	4.512	4.654	4.881	5.044	5.262	5.387
24	4.407	4.469	4.566	4.704	4.867	5.078	5.209
32	4.458	4.515	4.609	4.747	4.849	4.978	5.025
40	4.508	4.566	4.658	4.794	4.896	5.022	5.068
48	4.566	4.622	4.716	4.854	4.950	5.076	5.120
56	4.621	4.684	4.774	4.912	5.006	5.133	5.174
64	4.683	4.740	4.837	4.975	5.068	5.194	5.233
72	4.743	4.800	4.897	5.040	5.137	5.260	5.300
80	4.799	4.865	4.959	5.101	5.198	5.325	5.364
88	4.871	4.923	5.023	5.170	5.269	5.393	5.434
96	4.931	4.992	5.092	5.233	5.333	5.463	5.503
104	4.993	5.052	5.156	5.301	5.406	5.529	5.578
112	5.051	5.114	5.214	5.369	5.471	5.602	5.643

According to Table 1, Table 2, Table 3, Table 4, Table 5 and Table 6, the patch size of 25 × 25 and the search range of 96 × 96 exhibited the optimal performance. In the experiments, the computational time did not increase with the search range because the number of patch comparisons did not change. Therefore, the 25 × 25 patch size and 96 × 96 search range were tested on the entire Tsukuba Stereo Dataset. The average PSNR improvement for the 1800 frames was 9.764 dB.

**Table 5:** Mean PSNR improvement *vs.* search ranges.

Search Range														
Patch Size	8	16	24	32	40	48	56	64	72	80	88	96	104	112
3 × 3	5.015	5.518	5.726	6.011	6.393	6.010	6.040	6.304	6.391	6.372	6.188	6.321	6.243	6.084
5 × 5	5.819	5.682	5.896	5.879	6.083	5.972	5.586	5.666	6.323	6.232	6.173	6.204	6.233	6.140
7 × 7	5.215	5.603	5.611	5.698	5.885	5.852	5.850	5.871	6.151	6.267	6.115	5.993	5.625	6.147
9 × 9	5.638	5.581	5.643	6.099	6.085	6.240	6.532	5.905	6.260	6.615	6.557	6.535	6.223	6.436
11 × 11	5.632	6.016	5.947	6.126	5.781	5.741	5.824	5.726	6.123	6.224	6.412	6.684	6.564	6.755
13 × 13	5.750	5.966	6.060	5.965	6.128	6.428	6.405	6.199	6.175	6.522	6.628	6.528	6.715	6.901
15 × 15	6.018	6.133	6.136	6.253	6.403	6.362	6.344	6.347	6.281	6.935	6.529	6.592	6.708	6.372
17 × 17	6.396	6.396	6.691	6.565	6.715	6.765	6.780	6.918	7.002	7.114	7.043	7.142	7.128	7.097
19 × 19	6.533	6.533	6.504	6.483	6.418	6.688	6.691	6.539	6.910	7.024	6.933	7.067	7.211	7.046
21 × 21	6.386	6.386	6.499	6.770	6.738	6.656	6.907	6.849	7.145	7.609	7.584	7.502	7.448	7.430
23 × 23	6.264	6.264	6.222	6.543	6.672	6.721	6.745	6.749	6.835	6.921	6.981	7.127	7.193	7.348
25 × 25	6.612	6.612	6.612	6.701	6.760	7.150	7.216	7.232	7.135	7.121	7.343	7.385	7.426	7.466
27 × 27	6.700	6.700	6.700	6.541	6.707	6.696	6.850	6.974	7.175	7.138	7.074	7.150	7.198	7.219
29 × 29	6.795	6.795	6.795	6.683	6.794	6.569	6.673	6.684	6.715	6.818	6.839	7.020	6.867	6.851
31 × 31	6.701	6.701	6.701	6.710	6.703	6.623	6.702	6.769	7.005	6.982	7.057	7.016	7.032	7.014
Mean	6.098	6.192	6.249	6.335	6.418	6.432	6.476	6.449	6.642	6.793	6.764	6.818	6.788	6.820

**Table 6:** Mean SSIM after denoising vs. search ranges.

Search Range														
Patch Size	8	16	24	32	40	48	56	64	72	80	88	96	104	112
3 × 3	0.957	0.959	0.959	0.960	0.962	0.961	0.960	0.961	0.960	0.962	0.959	0.962	0.960	0.960
5 × 5	0.958	0.957	0.958	0.958	0.958	0.957	0.958	0.959	0.960	0.959	0.958	0.959	0.959	0.959
7 × 7	0.957	0.957	0.957	0.958	0.959	0.958	0.959	0.959	0.959	0.961	0.960	0.960	0.960	0.959
9 × 9	0.956	0.957	0.957	0.960	0.960	0.961	0.961	0.960	0.961	0.962	0.961	0.961	0.960	0.961
11 × 11	0.955	0.958	0.959	0.960	0.959	0.959	0.959	0.959	0.960	0.961	0.961	0.961	0.961	0.962
13 × 13	0.957	0.960	0.960	0.959	0.960	0.960	0.960	0.960	0.961	0.961	0.961	0.961	0.961	0.961
15 × 15	0.957	0.958	0.959	0.961	0.962	0.962	0.961	0.961	0.961	0.963	0.963	0.963	0.963	0.963
17 × 17	0.957	0.957	0.958	0.959	0.960	0.961	0.961	0.962	0.961	0.962	0.960	0.960	0.962	0.961
19 × 19	0.956	0.956	0.957	0.958	0.959	0.958	0.959	0.958	0.960	0.962	0.962	0.962	0.963	0.962
21 × 21	0.956	0.956	0.957	0.959	0.959	0.960	0.961	0.960	0.962	0.963	0.964	0.963	0.963	0.963
23 × 23	0.956	0.956	0.956	0.958	0.959	0.959	0.959	0.960	0.960	0.962	0.962	0.962	0.962	0.963
25 × 25	0.956	0.956	0.956	0.958	0.959	0.960	0.961	0.962	0.962	0.964	0.963	0.963	0.963	0.964
27 × 27	0.955	0.955	0.955	0.956	0.956	0.956	0.958	0.958	0.959	0.959	0.959	0.959	0.959	0.959
29 × 29	0.957	0.957	0.957	0.957	0.958	0.959	0.959	0.959	0.959	0.960	0.961	0.961	0.961	0.961
31 × 31	0.956	0.956	0.956	0.956	0.956	0.957	0.958	0.959	0.960	0.960	0.960	0.960	0.960	0.960
Mean	0.957	0.958	0.959	0.959	0.960	0.960	0.960	0.960	0.960	0.961	0.961	0.961	0.961	0.961

### 3.2. Experiments on a Real-World Scene

**Figure 7:**
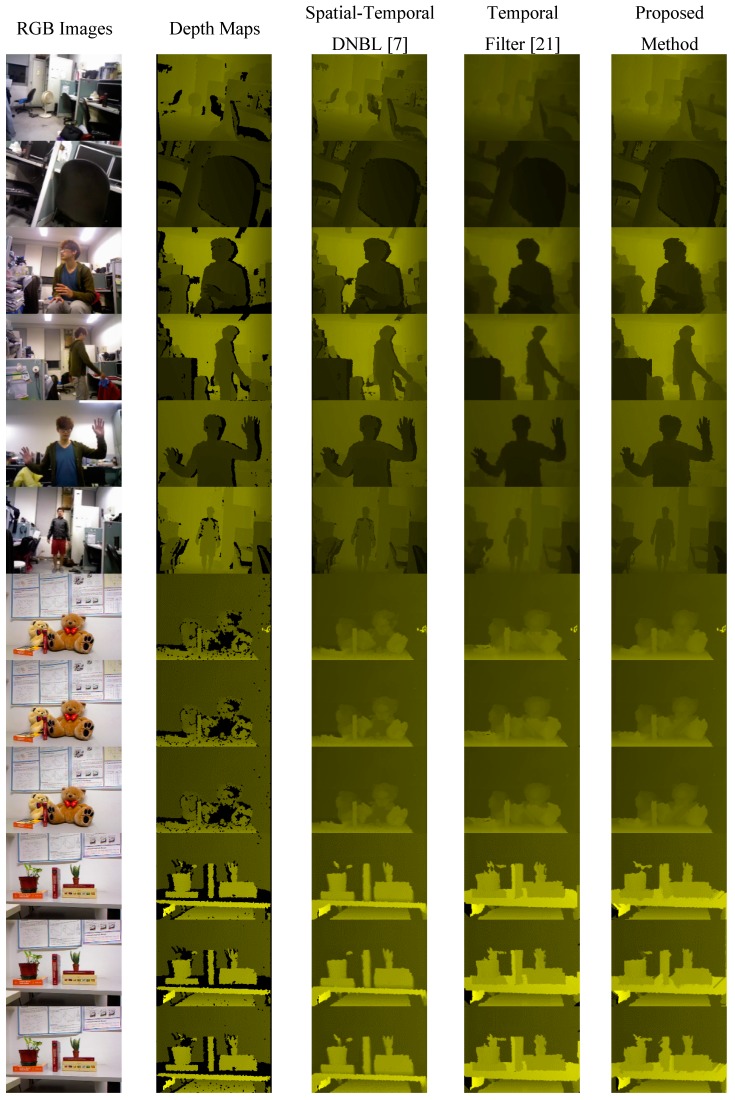
Real-world database and Milani’s Kinect dataset.

The real-world scene depth maps obtained using an Asus Xtion PRO LIVE depth camera and Milani’s Kinect dataset, the results of applying the proposed method and previously proposed methods are shown in Figure 7. The rows 7 to 12 of Figure 7 show the processed results of Milani’s Kinect dataset.

**Figure 8:**
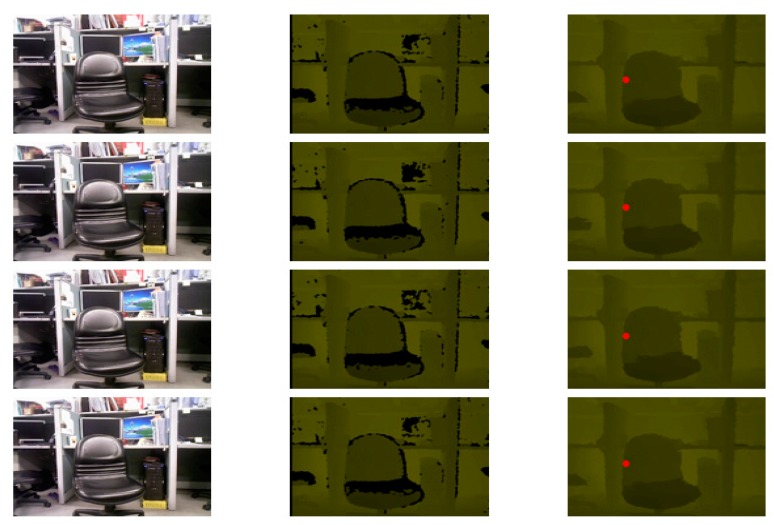
Temporally filtered images.

**Figure 9:**
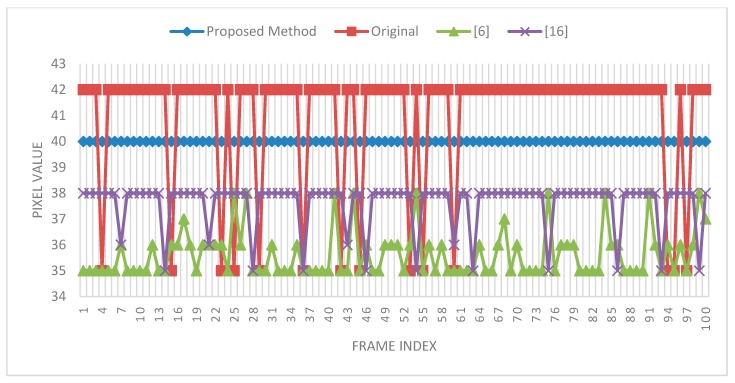
Temporal filtering results for 100 frames.

In addition to the experimental results for the spatial domain, the experimental results for the temporal domain are shown in Figure 8 and Figure 9. In Figure 8, there are three columns; the leftmost column shows the RGB images, the middle column shows the depth images, and the rightmost column shows the temporally filtered depth images. The red dot (256,180) in the rightmost column is the observation point selected to examine the variation in 100 frames. The experiment was performed with a search range of 40 and a patch size of 3 × 3. The experimental result is shown in Figure 9; the red square line denotes the original pixel variations and the diamond blue line denotes the filtered pixel variations in 100 frames.

## 4. Discussion

The proposed method successfully solved the problems of depth holes (outliers), inaccurate depth values, and temporal variations in pixel values. The experimental results demonstrated the high performance of the proposed method not only for the Tsukuba Stereo Database but also for real-world scenes. In the previous section, the experiments showed different results in different patch sizes, search ranges, temporal filtering, and comparisons with competitors.

As shown in Figure 5, Table 3 and Table 4, the application of the bigger patch size (25 × 25) resulted in the highest PSNR. The mean computational time was also less than that for the other small patch sizes; because a smaller patch fills fewer holes in the hole-padding process, it was necessary to apply the patch many times to fill the holes in the entire image. Applying larger patches resulted in shorter computational times (almost half the computational time taken for the patch size of 3 × 3) but higher PSNR values.

Table 5 shows the PSNR performance for different search ranges. In the modified TSS, as the search range increases, the PSNR improves considerably; this is because for larger search ranges, a larger number of reference patches are considered and selected, leading to improved PSNR performance. The application of smaller search ranges deteriorates the PSNR performance. However, Table 3 and Table 4 show that the computational time is shorter for smaller search ranges and larger for larger search ranges.

Table 2 shows the SSIM performance for different patch sizes and Table 6 shows the SSIM performance for different search ranges. In these two tables, the most SSIM values after denoising are greater than 0.95; that means the denoised depth maps are very similar to the ground truth maps in the visual scene.

Figure 4 shows comparisons of the PSNR and computational time between the proposed method and previously proposed methods. For the method proposed in [7], the computational time is considerably long and blurring reduces the PSNR performance. The method proposed in [21] is strongly dependent on RGB images and nonadaptive thresholds; therefore, when the scene changes, the PSNR performance decreases. Moreover, the method requires considerable time for computing the weighting metric for every single pixel. In Figure 7, our proposed method also shows better performance than [7,21] in the visual scene.

The patch size and search range strongly affect the PSNR performance and computational time. Although the computational times for the patch sizes of 9 × 9, 11 × 11, 13 × 13, 15 × 15, 17 × 17, 19 × 19, 21 × 21, 23 × 23, and 25 × 25 are extremely similar, 25 × 25 exhibits enhanced performance compared with others. Even if the computational times for 9 × 9, 11 × 11, 13 × 13, 15 × 15, 17 × 17, 19 × 19, 21 × 21, and 23 × 23 are considerably a little shorter than that for 25 × 25. Nevertheless, from Figure 5 and Figure 6, the patch size 25 × 25 could be realized the best mean PSNR improvements and mean SSIM performance. And the mean PSNR improvement starts to dip before patch size 17 × 17, but mean SSIM performances are similar for any patch size. Therefore, the patch size of 25 × 25 is the most favorable choice for obtaining the optimal balance between the PSNR performance and the computational time. Additionally, the search range of approximately 96 corresponds to a short computational time and high PSNR performance for the image size of 640 × 480 pixels. Summary, the patch size of 25 × 25 and the search range of 96 × 96 performed the optimal performance.

In Figure 9, the temporal filtering result shows constant and smooth variations (blue and diamond lines), unlike the original maps and the maps to which previously proposed methods were applied. The pixel values showing the smooth variation were also close to the original pixel values. In the experiment, the threshold was set to ten. However, in an optimal situation, an adaptive threshold should be used for different scenes to enhance the quality of depth images. Setting an appropriate threshold for different scenes to maintain the variation constant is challenging.

The proposed method showed superior PSNR performance compared with previously proposed methods. However, the computational time remains a problem in real-time applications, and future work should focus on developing methods for simultaneously achieving high performance and a short computational time.

## 5. Conclusions

This study presented a method for spatial and temporal denoising, removing depth noise, providing accurate depth values, and eliminating temporal variation in depth maps generated using RGB-D cameras.

The proposed method was applied to the well-known Tsukuba Stereo Database and a real-world scene database generated using an Asus Xtion PRO LIVE, and resulted in considerable improvement in the PSNR and SSIM of both datasets. The experimental results showed that in addition to PSNR and SSIM improvements, filtered depth maps of the proposed method were produced. The proposed method is based on the exemplar-based inpainting method proposed in [17], which has not been used for padding depth maps generated using RGB-D cameras. The most critical function of the proposed method is selecting the filling order of pixels around holes. The weighting scheme used is based on the isophote of the depth values forming a linear structure. The experiment results for the Tsukuba Stereo Dataset showed an improvement of 9.764 dB in the PSNR.

Increasing the PSNR and SSIM improvements and reducing the computational time for both the Tsukuba Stereo Dataset and real-time applications to obtain an enhanced quality of depth images are the focus of a study that is currently underway.
